# Bioactive IGF-I Concentrations in Children on GH Therapy

**DOI:** 10.1210/clinem/dgaf566

**Published:** 2025-10-14

**Authors:** Lea Vilmann, Jakob Albrethsen, Jørgen Holm Petersen, Stine Agergaard Holmboe, Peter Christiansen, Katharina Maria Main, Line Cleemann, Kristian Horsman Hansen, Jan Frystyk, Casper P Hagen, Anders Juul

**Affiliations:** Department of Growth and Reproduction, Copenhagen University Hospital—Rigshospitalet, Copenhagen 2100, Denmark; The International Research and Research Training Centre in Endocrine Disruption of Male Reproduction and Child Health (EDMaRC), Rigshospitalet, University of Copenhagen, Copenhagen 2100, Denmark; Department of Growth and Reproduction, Copenhagen University Hospital—Rigshospitalet, Copenhagen 2100, Denmark; The International Research and Research Training Centre in Endocrine Disruption of Male Reproduction and Child Health (EDMaRC), Rigshospitalet, University of Copenhagen, Copenhagen 2100, Denmark; Department of Growth and Reproduction, Copenhagen University Hospital—Rigshospitalet, Copenhagen 2100, Denmark; The International Research and Research Training Centre in Endocrine Disruption of Male Reproduction and Child Health (EDMaRC), Rigshospitalet, University of Copenhagen, Copenhagen 2100, Denmark; Section of Biostatistics, Faculty of Health and Medical Sciences, University of Copenhagen, Copenhagen 2100, Denmark; Department of Growth and Reproduction, Copenhagen University Hospital—Rigshospitalet, Copenhagen 2100, Denmark; The International Research and Research Training Centre in Endocrine Disruption of Male Reproduction and Child Health (EDMaRC), Rigshospitalet, University of Copenhagen, Copenhagen 2100, Denmark; Department of Growth and Reproduction, Copenhagen University Hospital—Rigshospitalet, Copenhagen 2100, Denmark; The International Research and Research Training Centre in Endocrine Disruption of Male Reproduction and Child Health (EDMaRC), Rigshospitalet, University of Copenhagen, Copenhagen 2100, Denmark; Department of Growth and Reproduction, Copenhagen University Hospital—Rigshospitalet, Copenhagen 2100, Denmark; The International Research and Research Training Centre in Endocrine Disruption of Male Reproduction and Child Health (EDMaRC), Rigshospitalet, University of Copenhagen, Copenhagen 2100, Denmark; Department of Clinical Medicine, University of Copenhagen, Copenhagen 2200, Denmark; Department of Growth and Reproduction, Copenhagen University Hospital—Rigshospitalet, Copenhagen 2100, Denmark; The International Research and Research Training Centre in Endocrine Disruption of Male Reproduction and Child Health (EDMaRC), Rigshospitalet, University of Copenhagen, Copenhagen 2100, Denmark; Molecular Endocrinology Unit, KMEB, Department of Endocrinology, Odense University Hospital, Odense M 5230, Denmark; OPEN Lab, Department of Endocrinology, Odense University Hospital, Odense 5230, Denmark; Department of Clinical Medicine, Faculty of Health Sciences, University of Southern Denmark, Odense 5230, Denmark; Department of Endocrinology, Odense University Hospital, Odense 5000, Denmark; Department of Growth and Reproduction, Copenhagen University Hospital—Rigshospitalet, Copenhagen 2100, Denmark; The International Research and Research Training Centre in Endocrine Disruption of Male Reproduction and Child Health (EDMaRC), Rigshospitalet, University of Copenhagen, Copenhagen 2100, Denmark; Department of Clinical Medicine, University of Copenhagen, Copenhagen 2200, Denmark; Department of Growth and Reproduction, Copenhagen University Hospital—Rigshospitalet, Copenhagen 2100, Denmark; The International Research and Research Training Centre in Endocrine Disruption of Male Reproduction and Child Health (EDMaRC), Rigshospitalet, University of Copenhagen, Copenhagen 2100, Denmark; Department of Clinical Medicine, University of Copenhagen, Copenhagen 2200, Denmark

**Keywords:** bioactive IGF-I, IGF-I, IGFBP-3, GH therapy, rhGH, LC-MS/MS

## Abstract

**Background:**

Monitoring IGF-I concentration is recommended during GH therapy in children. Supranormal levels of total IGF-I have raised concerns of long-term risks.

**Aim:**

To evaluate bioactive and total IGF-I in healthy and in GH-treated children and adolescents.

**Materials and methods:**

A reference population of 570 children (59% girls) from the Copenhagen Puberty Study III and 126 short children (36% girls) with GH deficiency (GHD) and other non-GHD conditions. We established pediatric, sex-specific reference ranges of serum concentrations of bioactive IGF-I (KIRA) and compared with total IGF-I and IGF-I/IGFBP-3 molar ratio (iSYS) and total IGF-I, -II, IGFBP-3, and acid labile subunit (liquid chromatography-tandem mass spectrometry) in a subgroup. Further, we compared IGF-I bioactivity with total IGF-I, IGFBP-3 (iSYS) and IGF-I/IGFBP-3 molar ratio during GH therapy.

**Results:**

Bioactive IGF-I increased with age in healthy children and correlated positively with total IGF-I and IGF-I/IGFBP-3 in healthy males (*r* = 0.61 and *r* = 0.57; *P* < .001) and females (*r* = 0.58 and *r* = 0.59; *P* < .001) and with IGF-I, BP-3, and acid labile subunit (liquid chromatography-tandem mass spectrometry) (*r* = 0.56, *r* = 0.29, and *r* = 0.38; *P* < .001). Bioactive IGF-I and IGF-II (*r* = -0.29; *P* < .001) correlated negatively. In 13% (17/126) of the patients, bioactive IGF-I was above +2SD, whereas total IGF-I was above +2SD in 25% (32/126) patients. Nine patients had both total and bioactive IGF-I above +2SD. In the non-GHD groups, bioactive IGF-I SDS were lower than IGF-I SDS (*P* = .015).

**Conclusion:**

Bioactive IGF-I was within reference ranges in the majority of GH-treated children. Monitoring bioactive IGF-I may help optimize GH dosing in specific patient subgroups.

In Europe, GH treatment is approved not only for short children with GH deficiency (GHD) but also for certain non-GHD conditions such as children born small for gestational age (SGA) without catch-up growth, Turner syndrome, Prader-Willi syndrome (PWS), Noonan syndrome, or SHOX haploinsufficiency. However, the growth response in these subgroups is very heterogeneous (1). Dosage of recombinant human (rh)GH is weight-based or dosed by body surface area. For decades, GH treatment of children with GHD has been monitored by the growth response and serum total IGF-I levels, which are maintained below the upper limit of the reference range (2). Normalization of growth and IGF-I levels through GH replacement reflects a well-established endocrine principle. However, these principles may not apply during GH therapy in short children with non-GHD conditions. In fact, 1 randomized controlled trial described that the majority of short children born SGA failed to grow when GH doses were titrated by serum total IGF-I (3). Thus, in non-GHD conditions, total IGF-I concentrations may not be an appropriate efficacy marker, but it may serve as a safety parameter to ensure that levels remain within reference range.

Sustained elevations in serum total IGF-I have prompted concerns regarding potential long-term risks, including cancer, but current evidence is predominantly from adult cohorts (4-6) and not from long-term surveillance studies of GH therapy. Although the follow-up period is limited, the Safety and Appropriateness of Growth hormone treatments in Europe (SAGhE) study found no effect of GH treatment on all-cause mortality nor cancer (7-9).

The IGF system controls metabolic and mitogenic responses in cells and thereby regulates embryonic growth and development as well as adult growth. IGF-II is an important regulator of fetal growth and levels are significant during fetal life, whereas postnatally the biological role of IGF-II remains uncertain (10). Levels of IGF-II drops right after birth and then increases until puberty and remains unchanged the remaining life (11). In the circulation, IGF-I and IGF-II compete for binding to IGF-binding proteins (IGFBP-1 to 6), with IGFBP-3 exhibiting the strongest affinity and binding more than 95% of IGF-I. The binary IGF-I/IGFBP-3 complex subsequently forms a ternary complex with the acid labile subunit (ALS) (12), thereby leaving only a small fraction of IGF-I being free and thus biologically available (13). Circulating IGF-I, IGFBP-3, and ALS are secreted by the liver in response to GH stimulation, whereas the regulation of IGF-II is less well understood, but its action is highly regulated by interaction with IGFBPs, in particularly IGFBP-3, which is also the major carrier of IGF-II in serum (10).

One way to estimate the biological activity of the circulating IGF system is by measuring the ability of serum to activate the IGF-I receptor (IGF-IR) in vitro (ie, bioactive IGF-I), as previously described (13). In this bioassay, buffer solutions containing recombinant human (rh)IGF-II is able to cross-react with the IGF-IR with an affinity being 12% of that of rhIGF-I (13). However, to which extent this can be translated to the in vivo situation in humans remains uncertain. For instance, in another study of children, total IGF-I correlated positively with the signal obtained by the bioassay, whereas serum total IGF-II did not correlate (14). For this reason, we often describe results obtained by the bioassay as “bioactive IGF-I.”

In a previous study, we demonstrated that markedly elevated total IGF-I concentrations during high-dose GH therapy in short SGA children were not reflected by supranormal bioactive IGF-I levels as measured by our bioassay (15). However, in children, GH treatment is also used in other indications than SGA, and therefore, the current study was intended to expand our previous findings to include short-stature children treated with GH for other reasons than SGA (ie, GHD, Turner syndrome, PWS, Noonan syndrome, and SHOX haploinsufficiency). We established a pediatric, sex-specific reference range of bioactive IGF-I (using our in-house KIRA) and evaluated bioactive IGF-I in a subgroup of healthy children against total IGF-I, IGF-II, IGFBP-3, and ALS measured by liquid chromatography-tandem mass spectroscopy (LC-MS/MS) and compared this with total IGF-I, and IGFBP-3 as measured by immunoassay (iSYS) in healthy children and patients with GHD and non-GHD disorders.

## Methods and Materials

### Reference Range for Bioactive IGF-I in a Cohort of Healthy Children

A subpopulation of 570 healthy children and adolescents (59% girls) from the ongoing cross-sectional, population-based Copenhagen Puberty Study III (COPUS III) served as reference population. Median (interquartile range [IQR]) age was for boys: 11.2 (9.1-13-3) and girls: 11.1 (9.2-14.4) years. Participants were evaluated by Tanner stage (16, 17), by either palpation of glandular breast or of testicular volume measured with an orchidometer. A single nonfasting blood sample was drawn from an antecubital vein between 8 Am and 12 Pm. Blood was centrifuged and stored at −80 °C until analyses of bioactive IGF-I and immunoassays for IGF-I, IGFBP-3, and ALS. In addition, a subgroup of samples was analyzed with the newly established LC-MS/MS method for quantifying IGF-I and IGF-II (n = 440), IGFBP-3 (n = 362), and ALS (n = 363).

### Children With Short Stature Receiving GH Treatment

In total, 126 patients (36% girls) with short stature were prospectively enrolled from our outpatient clinic at the Department of Growth and Reproduction at Rigshospitalet during 2024. Patients received GH therapy because of the following conditions: GHD (n = 73), SGA (n = 21), Turner syndrome (n = 7), PWS (n = 7), chronic renal insufficiency (n = 4), or other disorders (n = 14); the latter group was a mixed category of Noonan syndrome, SHOX haploinsufficiency, Silver-Russell syndrome, idiopathic short stature, and Charge syndrome. Median (IQR) age was 12.0 (9.0-13.4) years, for boys: 12.1 (8.7-14.1) and girls: 11.9 (9.9-13.0) years. Children received recombinant human GH at a median (IQR) dose of 26 (22-34) µg/kg/day, given as daily subcutaneous injections. As part of standard clinical evaluation, height, and weight were recorded, and a serum sample was drawn and stored at −80 °C until analyses. Bone age was determined from an X-ray of the left hand and calculated by BoneXpert; predicted adult height was calculated according to Bayley-Pinneau (18).

## Laboratory Measurements

*Total IGF-I* (RRID: AB_2861357) and *IGFBP-3* (RRID: AB_2895663) concentrations were determined using solid-phase enzyme-labeled chemiluminescence immunoassays (IDS-iSYS IGF-I and IDS-iSYS IGFBP-3; Immunodiagnostic Systems Ltd, Boldon, UK) on the IDS-iSYS Multi-Discipline Automated Analyser (IDS-iSYS, Pouilly-en-Auxois, France) at the Department of Growth and Reproduction, Rigshospitalet. The lower detection limits were 10 ng/mL for IGF-I and 80 ng/mL for IGFBP-3. Inter-assay coefficients of variation (CV) were below 7.2% for both analytes (19, 20).

*Bioactive IGF-I* (RRID: AB_3716599) was measured by an in-house IGF-I KIRA assay as previously reported (13, 21). In brief, the assays determine the ability of serum IGF-I to activate the IGF-I receptor in cells transfected with the human IGF-IR gene. After incubation of cells with serum at 37 °C, cells are lysed and the amount of phosphorylated (ie, activated) IGF-IRs is determined by specific ELISA. As we compare signals obtained from serum with a serial dilution of rhIGF-I (calibrated against the international World Health Organization standard), we are able to express the signal in serum samples as microgram per liter of IGF-I. The detection limit of the KIRA assay was 0.23 ng/mL, and the intra-assay CV of samples 14.2%. The long-term inter-assay CV of a control sample was 17.7%. As regards specificity, human insulin, insulin lispro, insulin aspart, and porcine proinsulin cross-react with <1%, whereas IGF-II cross reacts with 12%, when tested in buffer solutions containing the antigens without serum (13). However, the biological relevance of IGF-II activated IGF-IR remains uncertain. This is stressed by our previous observations in 342 children: whereas total IGF-I and bioactive IGF-I correlated positively in both prepubertal and pubertal children, as well as in all children, total IGF-II correlated positively with the KIRA assay signal in prepubertal children, but negative in pubertal children and therefore, showed no overall association (14). Therefore, we refer to the signal of the KIRA assay as “bioactive IGF-I.”

*IGF-I, IGF-II, IGFBP-3,* and *ALS* were analyzed in randomly selected samples from the healthy cohort based on a newly developed LC-MS/MS technique, at the Department of Growth and Reproduction, Rigshospitalet, as described in detail in Albrethsen et al 2024 (22). In brief, IGF proteins were extracted from serum and digested and selected tryptic peptides were quantitated by targeted proteomics. The long-term CVs for IGF-I, IGF-II, IGFBP3, and ALS were 7%, 25%, 7%, and 12%, respectively. The limit of detection for IGF-I, IGF-II, IGFBP3, and ALS were 6, 2, 90, and 300 ng/mL.

### Statistical Analyses

Age- and sex-specific SD scores (SDS) for IGF-I measured by iSYS were calculated from our reference data based on samples from 6459 healthy children, as previously published (23). Age- and sex-specific SDSs for bioactive IGF-I were calculated using our reference population of 570 healthy children. Reference ranges for all analytes were created using the Generalized Additive Model for Location, Scale, and Shape (GAMLSS) based on a Box-Cox distribution with age-varying coefficients. The GAMLSS model estimates a cross-sectional reference range and thus assumes that the data are cross-sectional. The data were summarized by 3 smoothed, age-dependent curves: L (age-dependent skewness), M (age-dependent median), and S (age-dependent coefficient of variation). Thus, age-related SDSs were calculated based on the GAMLSS model using the following equation: SD score = ((X/M)^L-1)/(L × S), where X is the measurement and L ≠ 0.

The IGF1/IGFBP3 molar ratio was calculated according to the formula as previously described by Friedrich et al (20): (IGF-I (ng/mL)×0.1307)/(IGFBP-3 (ng/mL)×0.0348).

**Figure 1.**
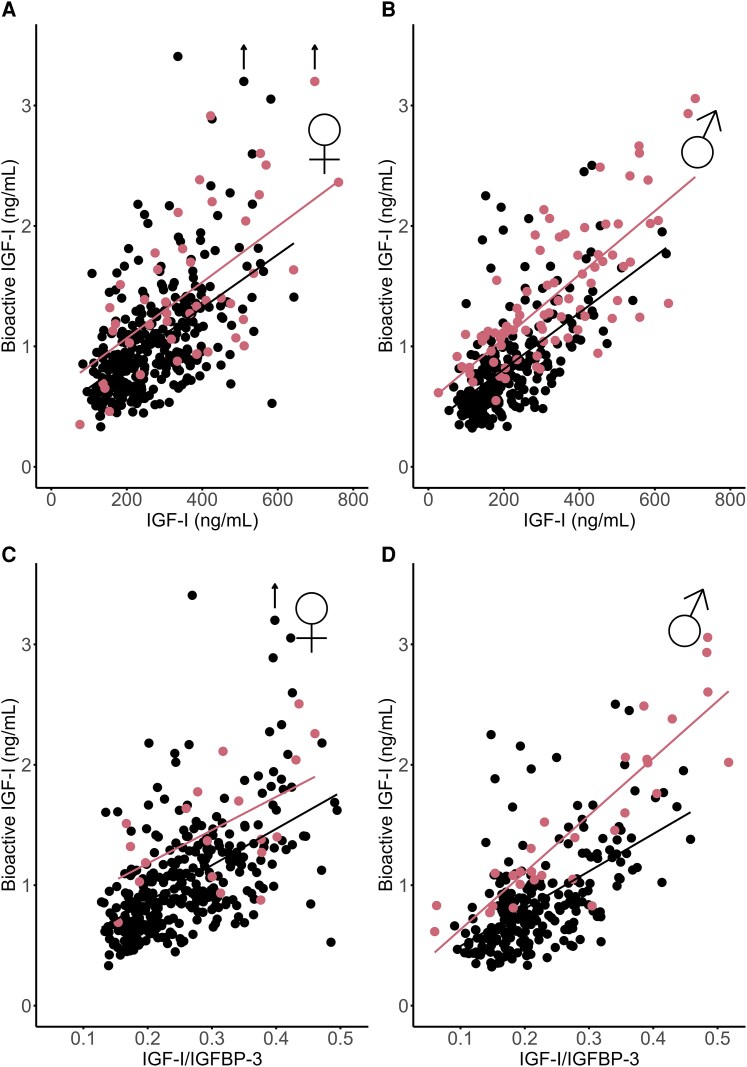
Bioactive IGF-I vs total IGF-I (A and B) and IGF-I/IGFBP-3 molar ratio (C and D) with regression lines in healthy controls (black) and in GH-treated patients (red).

**Figure 2.**
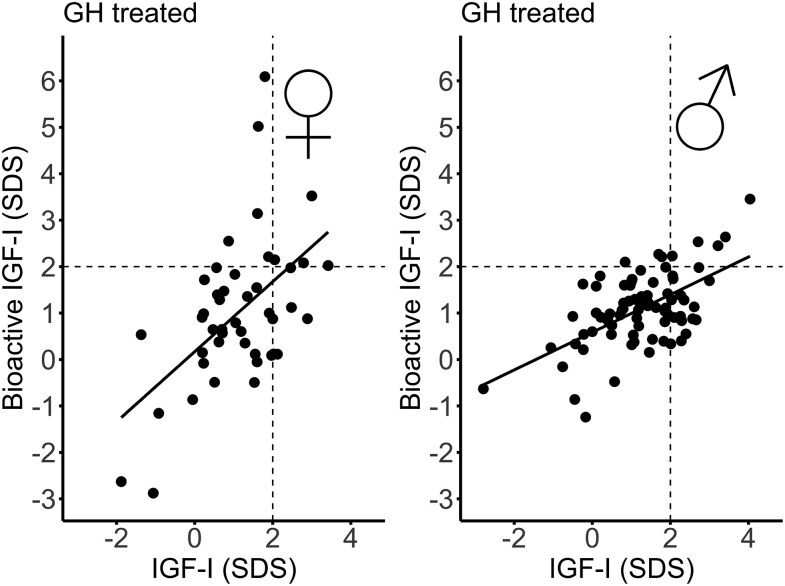
Bioactive and total IGF-I (SDS) with regression line in female and male GH-treated patients. Dashed black lines represents +2SD.

As not all data were normally distributed, results are presented as median (IQR) and analyzed using nonparametric tests as shown in Tables 1 and 2. However, when comparing SDSs in a correlation matrix (Table 3), Pearson correlation coefficients were used. Differences between sexes or between bioactive IGF-I SDSs and total IGF-I SDSs within patient groups were analyzed by Mann-Whitney *U* test. Spearman correlation coefficients were used to assess correlations between bioactive IGF-I and serum levels of IGF-I, IGFBP-3, IGF-I/IGFBP-3 ratio, and anthropometrics within patients. In Figs. 1 and 2, linear regression was applied to assess the relationships between selected variables of interest. *P* values < .05 were considered significant.

**Table 1.** Characteristics of healthy controls and patients

Healthy controls	n	All children	Female (n = 334)	Male (n = 236)	*P* value
Age (years)	570	11.2 (9.1-13.7)	11.1 (9.2-14.4)	11.2 (9.1-13.3)	.730
Weight (kg)	570	39.4 (29.5-53.8)	40.1 (29.5-54.3)	37.8 (29.7-53.6)	
Height (cm)	570	150.4 (137.5-164.9)	151.2 (138.1-164.5)	149.8 (136.9-168.3)	
Bioactive IGF-I (ng/mL)	570	0.87 (0.65-1.16)	0.93 (0.71-1.25)	0.78 (0.57-1.01)	**<**.**001**
Tanner stage 1			0.75 (0.61-0.93)	0.65 (0.49-0.87)	**<**.**001**
Tanner stage 2			0.99 (0.85-1.27)	0.76 (0.60-0.93)	**<**.**001**
Tanner stage 3			1.20 (0.98-1.51)	1.23 (0.84-1.57)	.747
Tanner stage 4			1.10 (0.84-1.36)	1.26 (0.85-1.62)	.460
Tanner stage 5			1.07 (0.79-1.35)	0.85 (0.64-1.07)	.**011**
IGF-I (ng/mL)	570	225 (166-325)	243 (183-346)	200 (147-279)	**<**.**001**
Tanner stage 1			172 (143-197)	152 (121-187)	**<**.**001**
Tanner stage 2			244 (215-304)	192 (147-237)	**<**.**001**
Tanner stage 3			333 (232-384)	338 (256-460)	.591
Tanner stage 4			349 (288-397)	334 (267-423)	.646
Tanner stage 5			332 (263-396)	297 (259-355)	.125
IGFBP-3 (ng/mL)	570	3782 (3351-4173)	3850 (3439-4221)	3675 (3244-4050)	**<**.**001**
**LC-MS/MS**			**n** *^a^*	**n = 203**	
IGF-I (ng/mL)	440	229 (168-315)	269 (190-362)	212 (160-260)	**<**.**001**
IGF-II (ng/mL)	440	172 (121-225)	158 (119-206)	190 (127-245)	**<**.**001**
IGFBP-3 (ng/mL)	362	2457 (2457-2825)	2502 (2185-2978)	2391 (2090-2761)	.**035**
ALS (ng/mL)	363	11 156 (9179-13379)	12 663 (10313-14782)	10 072 (8688-11960)	**<**.**001**

GH-treated patients	n	All children	Female (n = 45)	Male (n = 81)	*P* value
Age (years)	126	12.0 (9.1-13.4)	11.9 (9.9-13.0)	12.1 (8.7-14.1)	.627
Weight (kg)	126	36.5 (26.2-46.9)	38.5 (27.8-46.6)	35.8 (25.3-46.9)	.554
Weight SDS	126	−0.82 (-1.59 to -0.01)	-0.53 (-1.24 to 0.47)	-1.09 (-1.74 to -0.08)	.**033**
Height (cm)	126	142 (130-156)	141 (130-151)	142 (128-157)	.412
Height SDS	126	-1.31 (-2.06 to -0.71)	-1.57 (-2.22 to -0.79)	-1.14 (-1.96 to -0.62)	.250
PAH (cm)	120	169 (161-176)	160 (156-163)	174 (169-178)	**<**.**001**
GH dose (mg/kg/d)		0.026 (0.022-0.034)	0.025 (0.022-0.34)	0.027 (0.021-0.034)	.600
Duration of rGH (years)	126	3.83 (1.92-7.42)	3.42 (1.00-6.33)	3.83 (2.13-7.46)	.187
Bioactive IGF-I (ng/mL)	126	1.28 (1.05-1.76)	1.36 (1.04-1.93)	1.25 (2.13-7.46)	.420
Bioactive IGF-I (SDS)	126	1.05 (0.53-1.63)	0.89 (0.12-1.90)	1.11 (0.66-1.59)	.340
IGF-I (ng/mL)	126	334 (207-457)	369 (253-489)	307 (201-450)	.189
IGF-I (SDS)	126	1.26 (0.54-2.00)	1.19 (0.49-1.94)	1.30 (0.60-2.05)	.494
IGFBP-3 (ng/mL)	51	4043 (3303-4771)	4037 (3627-4444)*^b^*	4122 (2809-5033)*^b^*	.758
IGFBP-3 (SDS)	50	−0.56 (−1.12-0.42)	−0.66 (−1.19-0.25)*^b^*	−0.46 (−1.02-0.60)*^b^*	.411

Abbreviations: ALS, acid labile subunit; LC-MS/MS, liquid chromatography-tandem mass spectrometry; PAH, predicted adult height; SDS, SD score.

Data are presented as median (interquartile range). Comparison between the sexes was done by Mann-Whitney *U* test. *P* values < .05 (boldface) were considered significant.

^*a*^LC-MS/MS in females: IGF-I (n = 227), IGF-II (n = 227), IGFBP-3 (n = 150), and ALS (n = 151).

^*b*^IGFBP-3 (iSYS) for girls; n = 20 and males; n = 31.

**Table 2.** Characteristics of patient groups presented as median SDS (IQR)

Subgroups	n	GH dose (mg/kg/d)	Bioactive IGF-I (SDS)	IGF-I (SDS)	Spearman's rho
GHD	73	0.03 (0.02-0.04)	1.07 (0.46-1.67)	1.08 (0.31-1.82)	.442**
SGA	21	0.02 (0.02-0.04)	1.06 (−0.86-2.00)	1.88 (0.36-2.65)	.641**
Turner syndrome	7	0.02 (0.02-0.04)	0.12 (−0.49-2.08)	1.55 (0.51-2.05)	.821*
PWS	7	0.02 (0.01-0.02)	0.85 (0.65-1.13)	2.06 (0.70-2.61)	.536
CRI	4	0.03 (0.03-0.05)	1.24 (0.92-1.66)	1.41 (0.50-1.75)	—
Other diagnoses	14	0.03 (0.02-0.03)	0.85 (0.38-1.30)	1.41 (0.91-2.09)	.196
All diagnoses	126	0.03 (0.02-0.03)	1.05 (0.53-1.63)	1.26 (0.54-2.01)	.432**
All non-GHD diagnoses	53	0.02 (0.02-0.03)	1.00 (0.54-1.40)	1.55 (0.67-2.29)	.468**

Abbreviations: CRI, chronic renal insufficiency; GHD, GH deficiency; IQR, interquartile range; PWS, Prader-Willi syndrome; SDS, SD score; SGA, small for gestational age.

^*^Correlation is significant at the .05 level (2-tailed).

^**^Correlation is significant at the .01 level (2-tailed).

—not available due to limited power.

**Table 3.** Correlation matrix for all children treated with GH

	Bioactive IGF-I (SDS)	IGF-I (SDS)	IGFBP-3 (SDS)	Height (SDS)	Weight (SDS)	HV (SDS)	BMI (SDS)
**Bioactive IGF-I (SDS)**	1						
**IGF-I (SDS)**	.526**	1					
**IGFBP-3 (SDS)**	.255	.465**	1				
**Height (SDS)**	.028	.077	.065	1			
**Weight (SDS)**	.118	.254**	−.039	.595**	1		
**HV (SDS)**	.041	.180*	.023	.091	.061	1	
**BMI (SDS)**	.119	.249**	−.095	.137	.866**	.026	1

Abbreviations: BMI, body mass index; HV, height velocity; SDS, SD score.

Pearson correlations: *Correlation is significant at the .05 level (2-tailed); **correlation is significant at the .01 level (2-tailed).

Statistical analyses were performed using R Studio (version 2024.12.1) and SPSS (IBM Statistics, version 28).

#### Ethics

Research was conducted according to the Helsinki II declaration and approved by the Ethical Committee and by The Danish Protection Agency: H-19087825-96630 and P-2020-322 (COPUS III); H-22058254 and P-2023-14551 (Patients). COPUS III was registered by Clinical Trials: NCT04884620. Written informed consent was obtained from participants older than age 18 years or guardians of each child participating in the studies.

## Results

### Bioactive IGF-I

Bioactive IGF-I was positively correlated with total IGF-I in healthy males and females: *r* = 0.61 and *r* = 0.58 (both *P* < .001) (Fig. 1A and 1B) as well as with IGF-I/IGFBP-3 molar ratio; males: *r* = 0.57 and females: *r* = 0.59 (both *P* < .001) (Fig. 1C and 1D).

In addition, bioactive IGF-I correlated positively with IGF-I (*r* = 0.56), IGFBP-3 (*r* = 0.29), and ALS (*r* = 0.38; all *P* < .001) quantified by LC-MS/MS, whereas a negative association was seen between bioactive IGF-I and IGF-II (*r* = −0.29; *P* < .001). Figures are available in a digital research material repository (24, 25).

### Reference Ranges for Bioactive IGF-I

**Figure 3.**
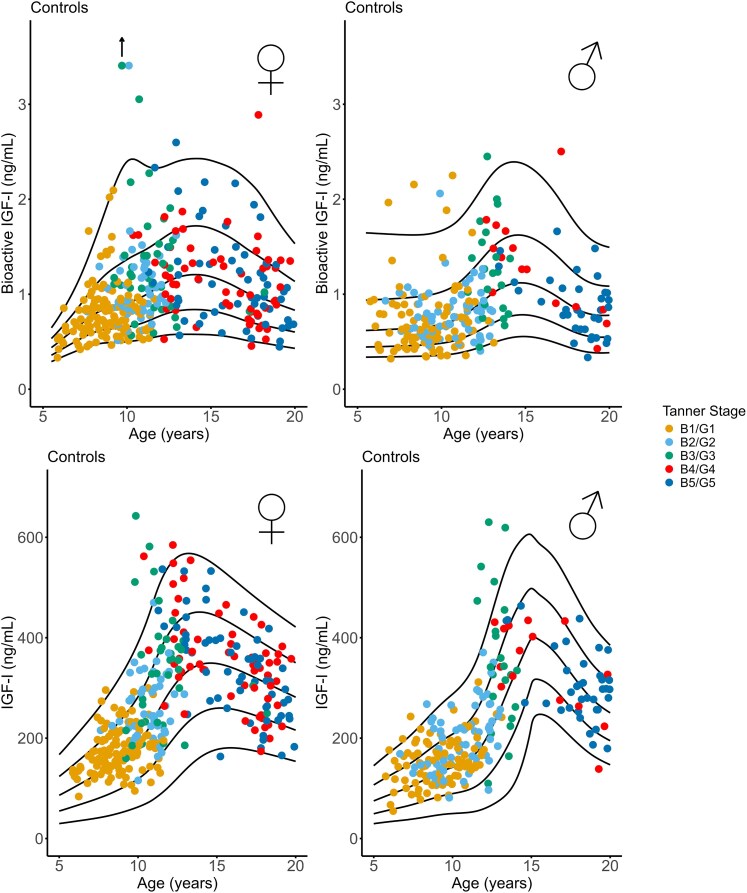
Bioactive and total IGF-I in healthy girls and boys according to Tanner stage of puberty. Black lines reflect mean, ±1SD and ±2SD.

Bioactive IGF-I increased during childhood and peaked at mid-puberty, Tanner stage B3/B4 in girls: 1.20 (0.98-1.51) (median [IQR]) ng/mL and G3/G4 in boys: 1.26 (0.84-1.62) ng/mL (Fig. 3 and Table 1). In general, levels of bioactive IGF-I were lower in boys as compared to girls (*P* < .001), also across Tanner stages (Table 1).

### Bioactive IGF-I and Total IGF-I in GH-treated Children and Adolescents

**Figure 4.**
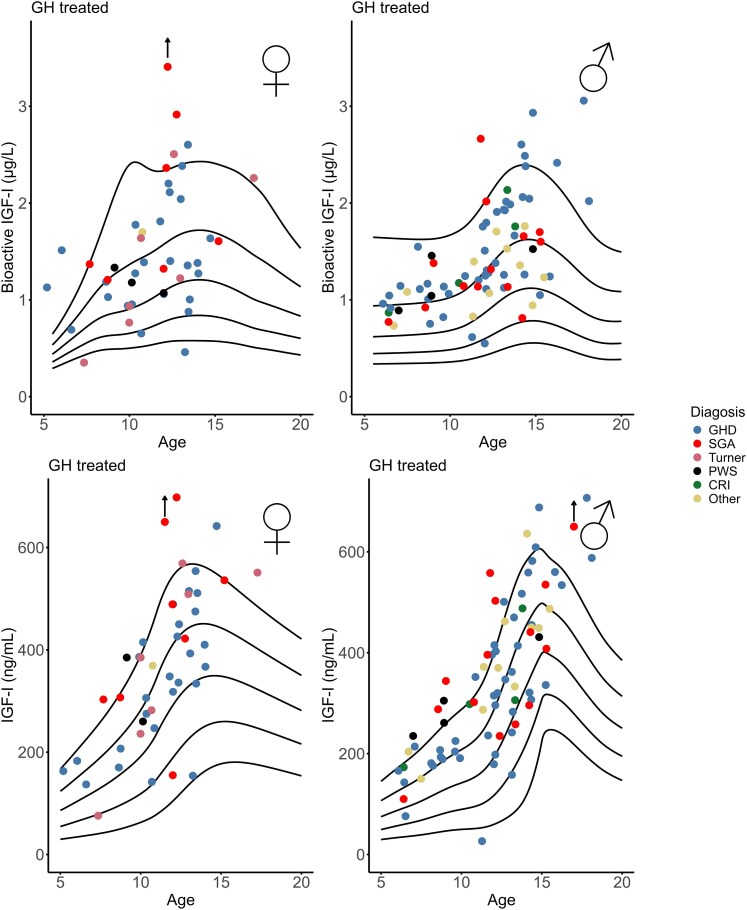
Bioactive IGF-I and total IGF-I in children receiving GH treatment by indication. Black lines reflect mean, ±1SD and ±2SD.

**Figure 5.**
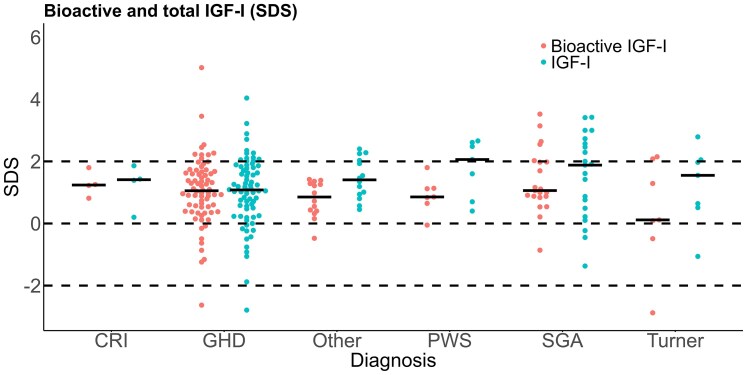
Bioactive and total IGF-I (SDS) by diagnosis. Gray horizontal bars represent median SD scores, dashed black lines represents mean and ±2SD.

Bioactive IGF-I was above +2SD in 13% (17/126) patients, whereas total IGF-I was above +2SD in 25% (32/126) patients (Figs. 2 and 4). In 9 patients, both bioactive IGF-I and total IGF-I were above +2SD. In 8 patients (6 with GHD and 2 born SGA), bioactive IGF-I levels were above +2SD, whereas total IGF-I remained below +2SD. Conversely, in 23 patients (9 with GHD, 6 SGA, 4 PWS, and 4 classified as “other” [including 3 with Silver-Russell syndrome, and 1 with Noonan syndrome]), bioactive IGF-I levels were below +2SD despite total IGF-I being above +2SD. In the non-GHD groups (SGA, Turner syndrome, PWS, chronic renal insufficiency, and “other diagnosis”), bioactive IGF-I SDS were lower than IGF-I SDS (*r* = 0.47, *P* < .001) (Fig. 5 and Table 2). There was no sex-specific difference of bioactive IGF-I serum levels in GH-treated patients (*P* = .36) (Table 1). In the GH-treated children, bioactive IGF-I SDS correlated with IGF-I SDS (*r* = 0.53, *P* < .001) (Fig. 2 and Table 3). Divided into subgroups, bioactive IGF-I and total IGF-I correlated positively within all patient groups except in the PWS and “other diagnoses” group (Table 2). Across subgroups, bioactive IGF-I correlated positively with IGF-I/IGFBP-3 molar ratio (*r* = 0.79, *P* < .001) (Fig. 1).

Bioactive IGF-I SDS correlated positively with total IGF-I SDS and weight SDS but there was no association with IGFBP-3 SDS, height SDS, height velocity SDS, nor body mass index SDS (Table 3). In addition, when repeating the analysis including only patients within ±2SD of body mass index SDS did not change the results.

## Discussion

This study compared serum levels of bioactive IGF-I using an in-house, validated (13) cell-based bioassay with measurements of the IGF system based on immunoassays and LC-MS/MS in a healthy population of children and adolescents. We present pediatric reference ranges for bioactive IGF-I for age, sex, and pubertal stage, and evaluated bioactive IGF-I in GH-treated patients with these reference ranges. We found that 13% of GH-treated children had bioactive IGF-I levels above +2SD, compared to 25% of patients who had elevated total IGF-I concentrations during standard GH therapy. This indicates that the qualitative responses of bioactive IGF-I and total IGF-I to GH treatment are different. Future studies are needed to evaluate if bioactive IGF-I can serve as a supplementary biomarker for GH dose titration, especially in non-GHD patients with poor growth response despite elevated total IGF-I concentrations.

Total IGF-I reflects the bound IGF-I as well as the free, bioavailable/bioactive IGF-I. The majority of IGF-I is bound to specific IGFBPs, particularly IGFBP-3, which form ternary complexes with ALS. These complexes prolong the half-life of circulating IGF-I but restricts its ability to activate receptors in target cells. Less than 1% of IGF-I is unbound and thus bioactive. As a result, total IGF-I may not reflect tissue-level activity of IGF-I.

To our knowledge, only 2 other studies have assessed bioactive IGF-I in children undergoing GH treatment; Wegmann et al (15) reported significant increases in bioactive IGF-I SDS after 1 year of GH therapy in children born SGA. Notably, although 68% had total IGF-I levels above +2SD, only 15% exceeded this threshold for bioactive IGF-I. In parallel, Bakker et al (26) found that nearly all patients had total IGF-I levels above +2SD, whereas only 1 of 40 children showed elevated bioactive IGF-I. They further concluded that in children with PWS, total IGF-I did not reliably distinguish between high and low IGF bioactivity.

IGF-I bioactivity has been evaluated in adults with GHD. In 2011 by Varewijck et al (27), bioactive IGF-I (KIRA) better reflected GHD than total IGF-I, thus insinuating the potential of bioactive IGF-I as a diagnostic tool. In 2015, they also demonstrated that during 12 months of GH treatment, changes in bioactive IGF-I did not parallel those in total IGF-I and remained subnormal in 40% of the patients (28).

Supranormal levels of total IGF-I during GH treatment are still a concern because of associations with a higher risk of adverse effects including increased risk of cancer later in life (4-6). However, most of these concerns are based on epidemiological studies of healthy middle-aged and elderly people, and do not necessarily apply to GH therapy during childhood.

In the follow-up SAGhE study, all-cause mortality and cancer risk was associated with the underlying diagnosis, but neither with the mean daily nor cumulative dose of rhGH (7, 9). In Kjaer et al (23), the cumulative lifetime exposure to total IGF-I and IGFBP-3 was examined in 6459 healthy participants and in 9 patients born SGA with 238 serum samples during GH treatment. Surprisingly, the mean lifetime exposure of total IGF-I in the GH-treated children was significantly lower compared to the mean lifetime exposure in the reference population. Although the results from both studies are reassuring, it is important to consider certain limitations, including a relatively short follow-up period and the absence of an untreated control group (the SAGhE study). The National Cooperative Growth Study, established to assess the safety and efficacy of GH treatment in children with growth disorders, evaluated 55 000 patients between December 1985 and January 2006, encompassing 192 354 patient-years of treatment (29). Certain patient groups with predispositions had a higher risk of adverse events and children previously treated with irradiation demonstrated an elevated risk in second neoplasms. Importantly, GH therapy was not associated with an increased risk of de novo leukemia, as confirmed previously (30-32). Overall, the safety profile of rhGH remains favorable with appropriate monitoring during and after therapy. To our knowledge, there are no long-term studies that have examined the lifetime effects of GH treatment during childhood, but the timing of which supranormal levels of total IGF-I are experienced may influence the risk of the development of malignancy. It is not clear whether high levels of total IGF-I should be a concern when bioactive IGF-I is within reference levels. However, the study design in the present study does not allow to evaluate bioactive IGF-I as a diagnostic tool, as well as treatment effect on growth nor the risk of long-term adverse effects.

In the current work, bioactive IGF-I and the IGF-I/IGFBP-3 molar ratio correlated positively but this is not always reproduced in other studies (33, 34). The diagnostic utility of serum IGF-I/IGFBP-3 molar ratio has been evaluated previously to be a useful clinical marker in the diagnosis of GHD (35). It is also considered an indirect biomarker of free/biologically active IGF-I to monitor GH treatment (36). Thus, we find it reassuringly that the IGF-I/IGFBP-3 molar ratio and the KIRA assay results are positively correlated. However, we believe the KIRA assay is a more direct estimate of the biological activity of IGF-I because it reflects real-time receptor activation.

There are limitations in this study. The estimation of bioactive IGF-I using the KIRA assay may overestimate the activity derived from IGF-I, as IGF-II is able to cross-react with 12%. However, as IGF-II is not GH dependent, this may be of less impact in this study. Furthermore, the specific treatment groups had relatively small sample sizes, which limits the statistical power. Another limitation in the present study is that repeated measurements were not included, and therefore we cannot assess whether bioactive IGF-I is a better or more accurate effect marker than total IGF-I during GH treatment. It is also noteworthy that our patients are treated with relatively modest GH doses compared to recommendations.

However, our study has several strengths, including the establishment of a contemporary healthy cohort of children and adolescents that form the foundation of bioactive IGF-I (KIRA) reference levels for future use. Moreover, we provide a large cohort of short stature children with different diagnoses, which has not been done before in relation to bioactive IGF-I. Finally, we present unique measurements from MS of IGF-I and 3 other proteins (IGF-II, IGFBP-3, and ALS) that influence the amount of biologically active IGF-I.

In conclusion, bioactive IGF-I increased throughout childhood and peaked around mid-puberty in both boys and girls. Among GH-treated patients, the prevalence of supranormal levels of bioactive IGF-I and total IGF-I varied across groups. Thus, our findings suggest that bioactive IGF-I may serve as a supplementary marker for GH titration, especially in non-GHD patients who exhibit poor growth response despite elevated total IGF-I.

## Data Availability

The datasets generated and analyzed during the current study are not publicly available. However, data may be made available from the corresponding author upon reasonable request and subject to approval by the relevant ethics committee and data use agreements.

## Abbreviations

ALS: acid labile subunit
COPUS III: Copenhagen Puberty Study III
CV: coefficient of variation
GAMLSS: Generalized Additive Model for Location, Scale, and Shape
GHD: GH deficiency
IGR-IR: IGF-I receptor
IQR: interquartile range
LC-MS/MS: liquid chromatography-tandem mass spectrometry
PWS: Prader-Willi syndrome
rh: recombinant human
SAGhE: Safety and Appropriateness of Growth hormone treatments in Europe
SDS: SD score
SGE: small for gestational age
